# Exploring general practitioners’ perspectives on the use and benefits of digital health applications for mental disorders in primary care: a mixed-methods study

**DOI:** 10.1186/s12913-026-14256-0

**Published:** 2026-02-28

**Authors:** Sandy Scheibe, Sandra Salm, Karola Mergenthal, Deborah Engesser, Esther Stalujanis, Susanne Singer, Pascal Kemmerer, Lena Dotzauer, Karen Voigt

**Affiliations:** 1 Department of General Practice, Faculty of Medicine Carl Gustav Carus, University of Technology Dresden, Löscherstraße 18, 01309 Dresden, Germany; 2 Institute of General Practice, Goethe University Frankfurt, Frankfurt am Main, Germany; 3 Institute of Medical Biostatistics, Epidemiology and Informatics (IMBEI), Epidemiology and Health Care Research, University Medical Center of the Johannes Gutenberg-University Mainz, Mainz, Germany; 4 Department of Consultation-Liaison Psychiatry and Psychosomatic Medicine, University Hospital Zürich, Zürich, Switzerland; 5 Clinical Psychology and Psychotherapy - Methods and Approaches, Department of Psychology, Trier University, Trier, Germany; 6 Comprehensive Cancer Center, University Medical Center Rostock, Rostock, Germany; 7 Department of Primary Care Research, Medical University Lausitz – CT, Cottbus, Germany; 8 Medical Psychology - Neuropsychology and Gender Studies and Center for Neuropsychological Diagnostics and Intervention (CeNDI), Faculty of Medicine and University Hospital Cologne, University of Cologne, Köln, Germany

**Keywords:** Digital health application, DHA-MD, Mental health, Mixed methods, General practice, Physicians, Primary care, Internet-based intervention

## Abstract

**Background:**

Worldwide, the high number of mental disorders poses challenges for healthcare systems. In 2020, digital health applications (DHA) were introduced in Germany as a novel type of healthcare financed by the statutory health insurance, aiming to detect, monitor, treat, or alleviate disease, injury, or disability. DHA for mental disorders (DHA-MD) intend to support outpatient care. However, there is limited evidence on general practitioners (GPs) perspectives on the use and benefits of DHA-MD in primary care.

**Methods:**

A mixed-methods study was conducted between January and October 2024, including a nationwide online survey and qualitative interviews with GPs from Germany. Sampling was conducted in collaboration with German practice-based research networks. The questionnaire and interview guide were developed by the study team using the domains of the Consolidated Framework for Implementation Research. Descriptive analyses of (un)suitable patient groups and treatment contexts for the use of DHA-MD in primary care were conducted. The interviews with GPs were analyzed using qualitative content analysis.

**Results:**

A total of 149 GPs participated in the survey and 12 GPs were interviewed. Most GPs reported that they would prescribe DHA-MD primarily to patients with prior positive experiences with DHA-MD (84.2%), aged 18-35 years (79.6%), with limited geographical mobility (78.6%), and when there is a need to bridge waiting times for psychotherapy (83.3%). In contrast, they would not prescribe DHA-MD to patients over 80 years (95.2%), under 18 years (87.0%), with multiple mental disorders (80.7%), and as primary treatment method (81.1%). In the interviews, GPs primarily viewed younger to middle-aged patients, with the necessary digital affinity and motivation to use the DHA, as well as with mild to moderate mental disorders as suitable. They reported to prescribe DHA-MD especially in waiting times and to support in-person care.

**Conclusions:**

GPs take into account patients’ individual skills, clinical condition, motivation, and likelihood of engaging with the DHA-MD when prescribing it. However, to avoid the systematic exclusion of certain patient groups, shared decision-making should be used to assess whether individual patients would benefit from using a DHA-MD. This assessment should be based on individual capabilities, such as digital literacy.

**Supplementary Information:**

The online version contains supplementary material available at 10.1186/s12913-026-14256-0.

## Background

With an estimated 970.1 million people affected worldwide in 2019, mental disorders account for a significant proportion of the global disease burden [1]. They are among the most burdensome health problems for those affected [1, 2], being associated with considerable limitations in quality of life and functional ability, often leading to incapacity to work, early retirement, and increased mortality risks [3–5]. In 2023, mental disorders accounted for 16.0% of all sick leave days in Germany and were the most common cause of early retirement, accounting for 41.8% of all cases [6, 7].

Despite the substantial need for adequate and accessible treatment options in mental health care, worldwide, the treatment coverage remains low, reflecting a substantial unmet need [8, 9]. In this context, the data showed that only 25.7% of individuals with mental disorders reported having accessed formal health care for mental health reasons. Even among individuals who access care, there is often a delay between symptom onset and the initiation of treatment [8–11]. In the European (EU) states, the median waiting time for psychotherapy exceeds two months [12]. In Germany, the recent psychotherapist act has potentially influenced the waiting times for the start of psychotherapy after initial consultation - with averages of 4.2 months before and 4.7 months after the reform [13].

Given the strained situation in the mental health care sector, mobile health (mHealth) applications have attracted considerable interest. In 2020, Germany introduced digital health applications (DHA) as a novel type of healthcare. Afterwards, other EU countries such as France, United Kingdom and Belgium have introduced approval procedures for DHA-equivalent health apps similar to the German model [14–16]. DHA are certified medical products, according to the EU Medical Device Regulation (MDR), primarily based on digital functions aiming to detect, monitor, treat, or alleviate disease, injury, or disability. For legal authorization as a medical device, DHA must demonstrate a positive healthcare effect (i.e., improvement of disease symptoms, quality of life, health literacy, or feeling better involved in the treatment) to be approved by the German Federal Institute for Drugs and Medical Devices (German, Bundesinstitut für Arzneimittel- und Medizinprodukte (BfArM)) [17]. To date (January 2026), 59 DHA can be reimbursed by statutory health insurance companies. Half of the existing DHA address patients with mental disorders (*n* = 30; 50.8%). These comprise apps for treatment of insomnia, depression, anxiety disorders or smoking cessation (digital health applications for mental disorders (DHA-MD)) [18]. For each DHA, the BfArM directory contains information on patient groups which are (un)suitable for its use, including relevant (contra)indications, gender, age group, and additional exclusion criteria that may preclude use (e.g., suicide risk). Additionally, some DHA require the provision of accompanying contractual medical services by physicians or psychotherapists, which can be reimbursed in addition to the prescription. Among the currently approved DHA-MD, three require such services, for instance, the individualization of the treatment program by physicians as suggested by the contents of the DHA-MD based on patients’ indication. The remaining 27 DHA-MD do not require any further physician involvement beyond establishing the diagnosis and informing the patient. The monitoring of treatment success and adherence by GPs is optional for these 27 DHA-MD and cannot be remunerated separately [18].

As reimbursement of DHA is only possible when prescribed by physicians or psychotherapists, these healthcare professionals play a key role in the implementation process [19–21]. In primary care, the general practitioner (GP) must determine implicitly or explicitly whether a specific DHA is appropriate for the individual patient [22]. Schroeder et al. [23] strengthened this gatekeeping role by introducing the concept of a second-order technology adoption process, meaning that a physician, as an upstream decision-maker, anticipates whether a patient - as the actual end user - would be able and willing to use the DHA. Only if this assessment is positive, the physician would consider offering the DHA [23]. According to the German Medical Association, the assessment should include whether the patient is actually able to use the DHA, considering usability, comprehensibility, digital affinity and literacy, as well as whether there are any comorbidities that would contraindicate the use of the DHA. Furthermore, GPs should decide on the appropriate duration of therapy (e.g., to bridge waiting times or as a long-term treatment), and whether other, potentially more (cost)-effective alternatives are available [24].

To date, there is little research on how GPs utilize these digital interventions for patients with mental disorders in general practice, especially in terms of which patient groups and treatment contexts they consider (un)suitable for the use of DHA-MD.

The studies by Schroeder et al. [23] and Dalhausen et al. [19], analyzing how physicians adopted mHealth apps in Germany, showed that physicians would prescribe DHA especially to young and tech-savvy patients. In contrast, older patients were considered unsuitable, as they are often perceived to lack both the interest and the ability to use electronic devices such as tablets or smartphones [19, 23]. Similarly, patients affected by social disadvantage - shaped by factors such as socioeconomic status, educational background, social capital, living conditions, and broader contextual influences (e.g., geographic or cultural) - may be systematically excluded due to anticipated barriers to use [25].

The qualitative study by Sarradon-Eck et al. [26], which analyzed GPs’ attitudes towards prescribing mHealth apps in France, found that GPs primarily view these apps as a complementary extension of routine care - particularly useful for promoting self-monitoring, enhancing patient education and improving treatment adherence - while emphasizing that they cannot replace direct physician-patient interaction. According to Weitzel et al. [27], which examined the current use of e-mental health by healthcare professionals in Germany, including DHA-MD, GPs mainly prescribe DHA-MD to support patients during waiting periods for psychotherapy.

These studies mentioned varied in the countries included and primarily focused on GPs’ prescription intentions, attitudes, and acceptance of DHA, rather than on how and why GPs use digital interventions in general practice. In addition, existing studies examine DHA across all approved indications, without focusing on those for mental disorders, which is of particular importance, as mental health conditions are among the most common reasons for consultation in general practice [28].

Our study addresses this gap by applying a mixed methods design to comprehensively analyze the use and perceived benefits of DHA-MD. Specifically, we aimed to investigate which patient groups and which treatment contexts GPs consider (un)suitable for the use of DHA-MD in primary care.

## Methods

This study was conducted in Germany. We applied a mixed-methods convergent parallel design in which quantitative and qualitative data were collected and analyzed separately, as described in detail elsewhere [29]. In a following step, triangulation of the results was conducted during the interpretation phase by comparing and contrasting the findings of both strands to identify areas of convergence, divergence, or complementarity [30, 31]. Both strands were given equal priority in the interpretation. To systematically integrate findings from qualitative and quantitative components, a joint display was developed that compares core results across data sources [32]. Reporting of the mixed-methods components follows the Good Reporting of A Mixed Methods Study (GRAMMS) framework [33].

### Online survey

#### Design and setting

Between January and October 2024, a web-based, anonymous survey was completed by practicing GPs (specialists in family or internal medicine) working in their own practices, as employed GPs, or as medical residents for general practice in GP practices. The survey link was disseminated nationwide via email through German practice-based research networks (‘DESAM-ForNet’ [34]), professional and trade associations, as well as presented at public events for GPs in Germany. Subsequently, the practice-based research networks forwarded the invitation to their affiliated GPs (snowball sampling approach). Due to this recruitment strategy, it was not possible to determine the exact number of GPs who received the invitation to participate.

#### Questionnaire

The questionnaire was developed by the study team using the domains of the Consolidated Framework for Implementation Research (CFIR) model, capturing barriers and enablers in the implementation of interventions [35]. Before implementation of the survey, we conducted a pretest of the questionnaire in November 2023 with six GPs who verified the relevance and completeness of the items. Based on the feedback of the participating GPs, the wording of one question was revised for improved clarity. The overall structure and methodology of the questionnaire was retained after pretest. No mandatory questions were used in the questionnaire to reduce respond burden and to allow GPs to skip questions they preferred not to answer. Missing values therefore reflect item non-response.

#### Demographic and practice characteristics

We collected sociodemographic information of the participants (i.e., gender, age, duration of practicing as GP) and characteristics of GP practices (i.e., type of practice [single/ group practice], location [urban/ rural], estimated number of treated patients per quarter [less than 500 patients-more than 4.000 patients], estimated average waiting time for access to psychotherapy in the region of the GP practice [less than 3 months-more than 6 months].

#### Perspectives on the use of DHA-MD

GPs were asked using 21 self-developed items which group of patients they would prescribe DHA-MD or not (e.g., different age groups, individual strain and situation). The treatment contexts in which GPs would prescribe DHA-MD or not were explored with five self-developed items (e.g., treatment priority, purpose). GPs assessed the suitability on a dichotomous scale (“No, I would rather not prescribe it” (0); “Yes, I would rather prescribe it” (1)).

#### Statistical analysis of the survey data

Quantitative data were analyzed using SPSS (Version 30; IBM Corp). Descriptive statistics were computed as mean values with standard deviations for metric-scaled variables and as percentages and frequencies for non-metric-scaled variables in order to describe the study sample. GPs’ response patterns regarding (un)suitable patient groups and treatment contexts for the prescription of DHA-MD were summarized as frequencies and percentages. Missing values were not imputed. Analyses were performed using pairwise deletion, such that participants with missing values were excluded only from the respective analyses, while being retained in all other analyses with complete data. This approach was chosen to maximize the use of available information.

### Qualitative interviews

#### Participants and procedures

Invitations for the telephone interviews were distributed via practice-based research networks in Saxony and Hesse (‘SaxoForN’ [36]), which subsequently forwarded the invitation by email to their affiliated GPs (snowball sampling). In addition, at the end of the online survey, information was provided for GPs about the opportunity to participate in a telephone interview on the topic of DHA. Interviews were conducted with all GPs who voluntarily registered via email to participate. Telephone interviews took place between July and October 2024 and were conducted by two researchers of the study team (SaS & SSch). Both interviewers were female and health scientists, whose methodological and content-related expertise complemented each other.

#### Telephone interviews

The semi-structured interview guide for GPs was developed using the domains of the CFIR framework [35]. It is presented in additional file 3. It started with an opening question, which transitioned to the following three key topics: experiences with DHA (overall; for DHA-MD), attitudes towards DHA-MD, implementation factors and conditions for the use of DHA-MD in outpatient care. To explore the perspectives on the use of DHA-MD and benefits in primary care, we asked GPs which patient groups cope (less) well with DHA-MD and what characteristics patients should have in order to be able to use a DHA-MD (e.g., indication, disease severity, personal characteristics, social condition, level of care need). If necessary, the interviewers asked further questions when it was natural to go more in-depth on the information the participants provided. Despite the interview guide, participants were encouraged to talk freely without too much interruption from the interviewers.

The interview guide was pretested by two GPs in December 2023. Feedback revealed that no substantial changes were deemed necessary in terms of methodology, structure or questions.

Theoretical saturation was assessed iteratively alongside data collection by the interviewers. Interviews were analyzed consecutively, and emerging codes and categories were continuously compared across interviews. Following established methodological recommendations on assessing data saturation in qualitative research [37], saturation was defined as the point at which no new categories emerged and additional interviews only contributed to the repetition or further elaboration of existing themes. In our study, saturation at the level of categories was reached after the analysis of the tenth interview. To confirm saturation and ensure sufficient heterogeneity of perspectives, two additional interviews were conducted, which did not yield substantively new insights. To ensure a transparent and consensual process, the assessment of saturation was discussed within the research team, and consensus was reached regarding the point at which theoretical saturation had been achieved.

The telephone interviews were audio-recorded and transcribed verbatim. To ensure data protection, all personal data were pseudonymized in the transcription process.

#### Qualitative content analysis

The interviews with GPs were analyzed using qualitative content analysis according to Mayring [38], following a structured and iterative procedure. As an initial step, a preliminary coding scheme for the interviews with GPs was developed deductively based on the previously developed interview guide and used as a set of main categories (see additional file 1). Two researchers (SSch & SaS) then analyzed the interviews independently with use of the software MAXQDA (Version 2020, VERBI Software). During this process, the coding framework was further refined inductively by developing subcategories derived from the interview material. Relevant meaning units were identified and assigned to appropriate subcategories, and illustrative quotations were selected. If further categories relevant to (un)suitable patient groups or treatment contexts emerged inductively during the analysis, these were incorporated into the results. After completing the initial coding of all interviews, the results were subsequently cross-compared, whereby disagreement was discussed until consensus was reached. If necessary, a third senior researcher (KV) was consulted. When several GPs expressed similar views on a topic, their statements were grouped and summarized in the results section to illustrate shared perspectives.

##### Reflexivity

The research team, drawn from health sciences and psychology, included members at different career stages (MSc, MPH, PhD), which brought varied perspectives to the study. The semi‑structured interview guide was developed based on the study objectives and refined through a participatory pretesting process with GPs.

The interviews were conducted by two researchers (SSch and SaS), both trained in qualitative research methods. The prior professional background of one interviewer (SSch) in the field of DHA was acknowledged as part of the reflexive process. No prior relationship existed between the interviewers and the participating GPs. Throughout data collection, efforts were made to establish a trusting interview atmosphere, and interviews were flexibly adapted to the individual flow of each conversation.

Data analysis was conducted iteratively by two researchers (SSch and SaS), with category development and coding discussed regularly within the research team to promote reflexivity and reduce individual interpretive bias. After the interviews, the interviewers shared their research interests with participants (‘debriefing’). Confidentiality and anonymity were assured throughout the study to foster open responses and to minimize social desirability bias.

## Results

### Online survey

#### Characteristics of the survey participants

A total of 149 participants completed the questionnaire, of whom 47.7% (71/149) were male and 52.3% (78/149) were female. As shown in Table 1, the mean age of the respondents was 50.7 years (SD=10.5 years). The mean work experience of GPs was 15.2 years (SD=9.9 years). Most respondents were practice owners (111/149, 74.5%) and located in urban areas (122/148, 82.4%) with various community sizes.

**Table 1:** Description of the survey participants

Categorial variables		
	Total *N*	Sub-Total *N* (%)
Gender	148	
Male		71 (47.7)
Female		78 (52.3)
Position in practice	149	
Practice owner		111 (74.5)
Employed		31 (20.8)
Clinical resident		7 (4.7)
Type of practice	145	
Single practice		74 (51.1)
Joint practice		71 (48.9)
Practice location	148	
Large city (> 100.000 inhabitants)		50 (33.8)
Medium sized city (20.000-100.000 inhabitants)		35 (23.6)
Small city (5.000–20.000 inhabitants)		37 (25.0)
Rural community		26 (17.6)
Communication with patients & colleagues in practice	141	
Digitized		26 (18.4)
Half digitized/ half paper-based		62 (44.0)
Paper-based		53 (37.6)
Treated patients per quarter	145	
< 1000 patients		18 (12.2)
1000–1999 patients		75 (50.6)
2000–2999 patients		33 (22.3)
> 3000 patients		19 (12.9)
Average waiting time for psychotherapy in the region	148	
< 4 wks		1 (0.7)
1 to < 2 mo		10 (6.8)
2 to < 3 mo		25 (16.9)
3 to 6 mo		74 (50.0)
> 6 mo		38 (25.7)
*Numerical variables*		
	Total N	M (SD)
Age, years	149	50.7 (10.5)
Duration of GP practice, years	148	15.2 (10.0)

Legend: wks= weeks; mo=months

#### Perspectives on the use and benefits of DHA-MD

##### Age group

**Fig. 1:**
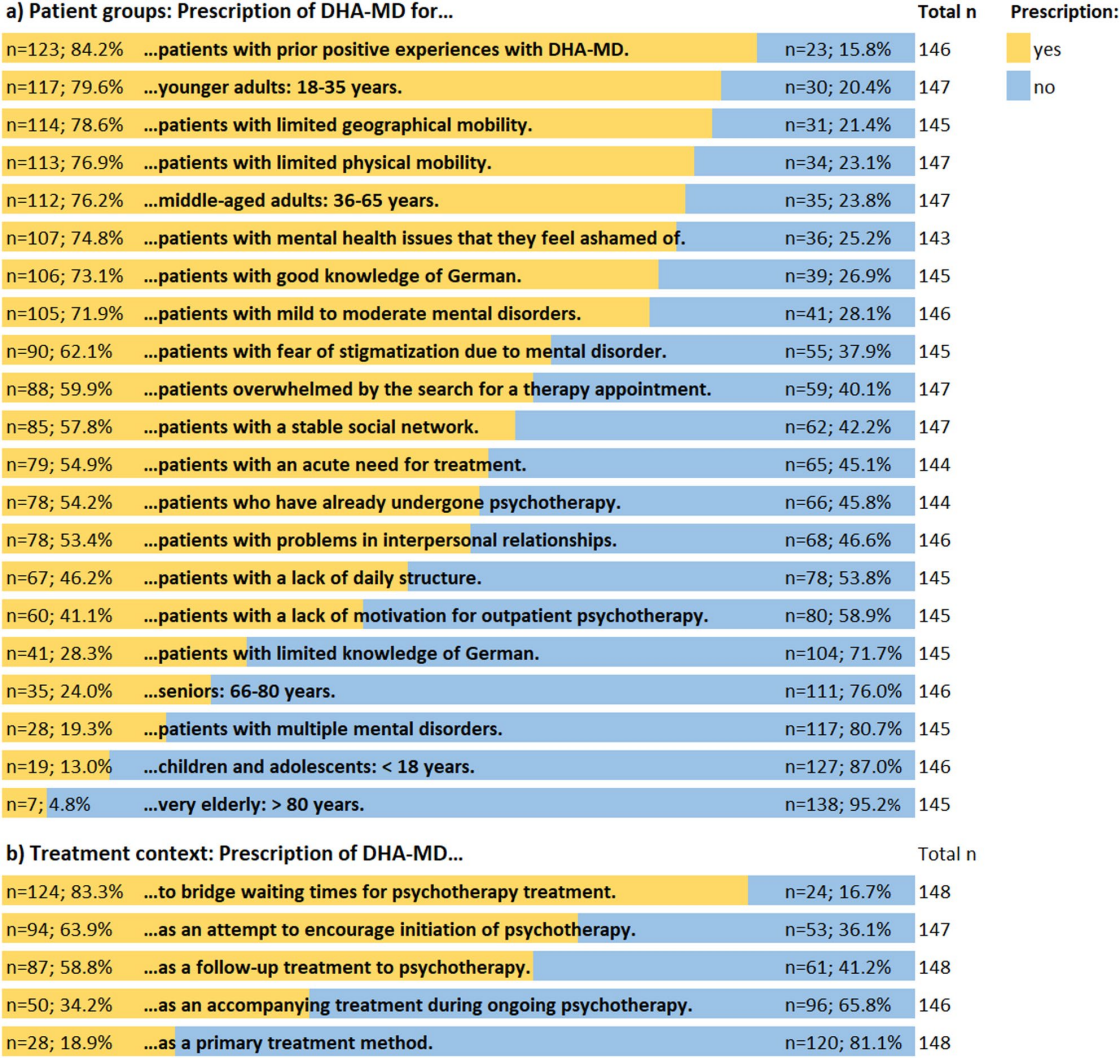
Responses patterns of GPs on the use and benefits for DHA-MD; figure based on Engesser et al. [39]

With regard to different patient age groups, there was an agreement of GPs to *rather prescribe* DHA-MD to patients aged 18-35 years (79.6%), and 36-65 years (76.2%) (see Fig. 1). For patients under 18 years (87.0%), as well as for those between 66 and 80 years (76.0%) and over 80 years of age (95.2%), most of participating GPs considered the use of DHA-MD *unsuitable*.

##### Clinical status and therapy, psychosocial burden and social resources

When considering individual strain and patient situation, most of the respondents reported that they would *rather prescribe* DHA-MD to patients who have already had positive user experiences with DHA-MD in the past (84.2%), to patients with mental health issues they feel ashamed of (74.8%), as well as to patients with limited geographical (78.6%) and physical mobility (76.9%). DHA-MD were predominantly *not considered suitable* for patients with multiple mental health conditions (80.7%).

##### Treatment priority and purpose

The majority of GPs reported that they would *rather not prescribe* DHA-MD as a primary treatment method (81.1%). However, most GPs indicated that they *would prefer to prescribe* DHA-MD in the treatment context of bridging waiting times for psychotherapy (83.3%).

### Telephone interviews

Interviews were conducted with 12 GPs and varied in duration between 18 and 38 min (average duration: 25 min).

#### Characteristics of the interview participants

A total of 12 GPs participated in the interviews, of whom 75.0% (9/12) were female and 25.0% (3/12) were male. The mean age was 46.2 years (SD=8.7), and the mean work experience was 9.5 years (SD=8.3). All participants had prior experience prescribing DHA.

#### Perspectives on the use and benefits of DHA-MD

The qualitative content analysis yielded three main categories and 11 subcategories, which are presented in additional file 1. The results for each subcategory are described below, accompanied by illustrative quotes from the participating GPs.

##### Indication area

The indications that GPs considered (un)suitable for the use of a DHA-MD varied greatly. There was a group of GPs that considered anxiety or panic disorders suitable for the use of DHA-MD. This was justified as patients may fear in-person therapy sessions or find the journey to a psychotherapy practice burdensome, which, in their opinion, could ease access to and encourage the use of a DHA-MD. In addition, GPs noted that therapeutic contents - such as relaxation techniques in cases of anxiety and panic disorders - can be effectively and sustainably reinforced through the use of a DHA-MD.

*[…] Because someone who has an anxiety or panic disorder is actually fit. Or*,* that’s not true*,* but many of these people […] have a lot of drive*,* right? They’re not listless and constantly tired and so on*,* so they can deal with something like this. And I think it’s important to repeat and reinforce these relaxation exercises after therapy*,* so to speak. And maybe that makes sense with an app like this. (Sample quote 1*,* GP_FFM04)*

The responses of GPs regarding the prescription of DHA-MD to patients with depressive disorders were heterogeneous. Some GPs highlighted the use of DHA-MD particularly suitable for patients with depressive disorders, as the reduced drive could make it easier for them to engage with the contents of an app rather than schedule and attend in-person psychotherapy sessions. Others, however, argue that the reduced drive may compromise adherence and that its use could further reinforce patients’ social withdrawal.

Other indications mentioned by individual GPs as suitable for the use of a DHA-MD included adjustment disorders, stress-related complaints, burnout, psychosomatic conditions, substance use disorders (e.g., smoking and risky alcohol consumption) as well as insomnia. In contrast, the following indications were reported by individual GPs as unsuitable: psychotic disorders with presumed suicidality, substance use disorders (specifically drug addiction), and post-traumatic stress disorders.

##### Clinical status and therapy experience

According to the disease severity for which the use of a DHA-MD was considered (un)suitable, two different groups of GPs emerged in the analysis. The first group of GPs reported the use of DHA-MD as suitable only for patients with mild to moderate mental disorders, arguing that a certain degree of clinical stability is necessary, especially since GPs often report that they are not completely familiar with the specific app contents. They mentioned that patients with mild to moderate mental disorders may be more likely to develop the motivation needed to engage with the contents of a DHA-MD. Additionally, this group of GPs reported that patients with severe mental disorders (most frequently mentioned severe depression) may be unsuitable for DHA-MD use, due to a lack of drive and insufficient daily structure to use the app comprehensively. However, a second group of GPs reported that they would prescribe DHA-MD to patients with severe mental disorders, as they believed these patients often lack the motivation to attend in-person psychotherapy sessions and perceive the barrier to using an app as lower. In this context, GPs highlighted that patients should be in a stable condition without signs of acute deterioration or suicidality.

*You can treat every [level of disease severity]. It depends on how they are managed*,* right? If you have someone with a severe condition who is well managed*,* then you can [prescribe it] … The thing is*,* it’s actually a counseling offer. (Sample quote 2*,* GP_DD01)*

In the case of patients with chronic mental health conditions, GPs reported that they would be less likely to prescribe a DHA-MD, as they tend to perceive DHA-MD only as short-term interventions.

##### Treatment priority and purpose

Individual GPs reported that they consider patients’ individual circumstances and level of care need when deciding whether to prescribe a DHA-MD. In particular, they mentioned being more inclined to recommend a DHA-MD for patients who struggle to access psychotherapy. In contrast, they noted that if patients had previously had positive experiences with in-person psychotherapy, they might be less inclined to prescribe a DHA-MD. This was justified by stating that patients who have already undergone psychotherapy - especially cognitive behavioral therapy - are often familiar with the fundamental coping strategies and techniques typically provided in such apps. In addition, some GPs emphasized that a certain level of digital autonomy is required for the use of DHA-MDs, and expressed reservations about prescribing them to patients who rely on assistance for daily tasks (e.g., patients in nursing homes) or lack the ability to operate a smartphone independently.

*Well*,* I would of course explicitly not prescribe it to people who can no longer manage the [use] on their own. That means everyone who is now in a nursing home*,* or*,* well*,* not necessarily a nursing home in general*,* but if someone*,* for example*,* needs assistance in order to take part in something like this*,* then that would already be quite a high barrier for me […] because they always need a second person to set everything up and so on. That means people have to be able to operate their smartphone independently. (Sample quote 3*,* GP_FFM02)*

Moreover, they reported to prescribe DHA-MD primarily in the treatment context to bridge patients waiting times for psychotherapy as well as an initiation and support of in-person care.

##### Individual patient characteristics

GPs highlighted a wide range of personal characteristics they considered necessary for the use of a DHA-MD, as well as traits they perceived as barriers to its use. In the analysis, GPs’ responses could be grouped into three categories: personality traits, age groups, as well as digital literacy & usage habits. These categories are described in detail below.

##### Personality traits

A first group of GPs stated that patients’ motivation and self-initiative are crucial for using a DHA-MD. They highlighted that patients should be willing to actively work on their health, should have a high level of compliance, and feel sufficiently distressed to seriously strive for a change in behavior.

*“So [the patient] said it was really quite exhausting [to use the DHA-MD]. And it only works for very compliant patients*,* one has to say. So*,* for people who don’t stick with it well*,* it’s difficult.” (Sample quote 4*,* GP_DD02)*

Furthermore, they reported that patients should be open-minded to this novel type of treatment. In contrast, GPs reported that they are less likely to prescribe a DHA-MD to patients who, in their view, lack the necessary intrinsic motivation and would need to be regularly reminded or scheduled for follow-up appointments.

##### Age groups

A second group of GPs highlighted that the prescription of a DHA-MD is more suitable for younger to middle-aged patients (range 20-60 years) as this age group is perceived more digitally skilled and comfortable with smartphones. In contrast, older adults or seniors are considered less suitable for the use of a DHA-MD due to a limited digital competence and, according to GPs, a preference for personal contact with a physician or psychotherapist. The interviews also showed that GPs would be willing to prescribe DHA-MD to patients under the age of 18 if future legal regulations were to permit it.

##### Digital literacy and usage habits

A third group of GPs emphasized that digital affinity is an essential prerequisite for patients to use a DHA-MD. Patients were perceived to need a general interest in and openness to digital technology as well as a basic technical understanding to use mobile devices, such as smartphones. In addition, they emphasized that patients should have access to a suitable device and be able to independently carry out steps, such as downloading the app or redeeming the activation code before the use. Previous positive experiences with other apps were also mentioned as potentially promoting factors for the use of a DHA-MD.

*“Of course*,* we’re talking about a technical product. You don’t necessarily have to be technically savvy in any particular way*,* but at least not dismissive toward technology. Most people at least have the basic requirements and somehow a smartphone or a tablet or whatever.” (Sample quote 5*,* GP_DD08)*

Other personal characteristics that individual GPs considered less suitable for the prescription of a DHA-MD included anxious personality traits, as in their opinion, the use of such apps could potentially exacerbate symptoms - particularly due to concerns about data privacy. In addition, patients with excessive or problematic smartphone use, as well as those facing language barriers that might hinder effective app use, were also seen as less suitable for a DHA-MD.

#### Integration of findings

A joint display integrating quantitative and qualitative findings on GPs’ perspectives on the use and benefits of DHA-MD is provided in additional file 2.

## Discussion

The aim of our study was to analyze GPs’ perspectives on the use and benefits of DHA-MD for patients with mental disorders in primary care. Building on this, we provide the first insights into GPs’ assessments of which patients they consider (un)suitable for DHA-MD prescription, beyond the formal eligibility criteria set out in the BfArM directory. In making these judgments, GPs particularly take into account patients’ clinical condition as well as personal characteristics and individual skills.

### Perspectives on the use and benefits of DHA-MD

#### Age group, personality traits, digital literacy and usage habits

With regard to different age groups, our quantitative findings show that GPs would primarily prescribe DHA-MD to patients aged 18 to 65 years, which is consistent with the German DHA report of the National Association for Digital Healthcare indicating an average user age of 42 years [40]. In the interviews, GPs attributed this preference to the assumption that this age group possesses the necessary digital affinity, openness, and motivation to engage with the contents of a DHA-MD. This is in line with the findings of Brecher et al. [41], analyzing DHA use in the German primary care setting, stating that 72.9% of GPs take patient-related factors such as age, digital affinity, and motivation into account before prescribing it. This was strengthened in our interviews with GPs, showing that patients need to be interested in digital technology and possess basic technical skills, as well as access to compatible devices, to be suitable for the use of a DHA-MD. In this context, GPs emphasized that without sufficient motivation and compliance, patients might not engage regularly with the app contents. Adherent use is essential not only to achieve therapeutic success but also from the perspective of the overall budget of German health insurance funds. Given limited healthcare resources and the relatively high costs of DHA-MD, the prescription should be considered carefully [42]. At the same time, the cost-effectiveness of DHA-MD is complex and to date insufficiently investigated [43]. A recent study analyzing the cost-effectiveness of mHealth apps for depression indicates that DHA-MD may not directly reduce overall healthcare costs but can contribute to the improvement of patient care and public health outcomes by increasing quality-adjusted life years [44]. By offering nationwide access, DHA-MD may help reduce existing care gaps and bridge waiting times for psychotherapy, thereby potentially preventing symptom deterioration, or poorer remission outcomes [45].

However, a sound assessment of patients’ digital affinity might be difficult to carry out in the limited time available during routine consultations, and physicians’ perceptions may not always correspond to patients’ actual abilities. It can be assumed that age serves as a surrogate marker for digital affinity, meaning that GPs perceive older people as less digitally competent, while younger people are considered more digitally skilled [19, 25, 46, 47]. These considerations are closely linked to social inequalities in healthcare and systematic differences in digital participation (def. “digital divide”). Previous research indicates that the utilization of digital health interventions is strongly shaped by patients’ social determinants, with younger, more educated, and higher‑income individuals being more likely to benefit, while older adults and socioeconomically disadvantaged individuals are less likely to do so [25, 48].

In this context, shared decision-making may help prevent the systematic exclusion of certain patient groups. Actively discussing patients’ preferences, expectations, and concerns regarding the use of DHA‑MD, as well as jointly selecting the most suitable DHA-MD from the available options, may enhance patients’ motivation, acceptance, and adherence [49].

Beyond shared decision-making, the provision of structured remote support and assistance, combined with efforts to strengthen patients’ knowledge and confidence in using digital health services, may further reduce digital inequalities [50]. Such support could be operationalized through the implementation of ‘digital navigators’ [51], specially trained medical assistants who provide ongoing guidance and support to patients when using the app and support healthcare professionals in selecting suitable apps. To avoid creating additional interfaces and to enable more holistic care, such support could be anchored within GP practices and integrated into routine primary care workflows. This role could also be taken on by existing practice staff (e.g. a medical assistant already working in the GP practice) who could receive additional training for this purpose.

In addition, strengthening digital health literacy at the population level represents another promising approach to reducing systematic differences in digital participation. A scoping review on digital health technologies and inequalities suggests that investments in adult education, community‑based digital peer‑learning, and programs delivered by organizations trusted by marginalized groups can significantly improve digital skills and participation [50]. At the national level, initiatives such as the National Health Service program “Widening Digital Participation” in England illustrate how collaborations with libraries and community organizations in socially deprived areas can improve digital literacy and promote equity [52].

The reluctance to prescribe DHA-MD to children and adolescents under 18 years, as clearly indicated in the online survey, may be explained by the fact that DHA-MD are only approved for adults [18]. However, GPs emphasized that they would consider prescription, given the perceived high level of digital affinity among this younger population, if the legal framework would allow it.

#### Psychosocial burden and social resources

When taking into account patients’ psychosocial burden and social resources, GPs expressed heterogeneity with regards to prescribing DHA-MD to individuals experiencing difficulties in interpersonal relationships and to those with a stable social network. One possible explanation could be that GPs may assume that patients with interpersonal problems require personal support and might further withdraw through the use of a DHA-MD. In contrast, the heterogeneous responses concerning patients with a stable social network may be due to the assumption that these individuals already benefit of sufficient support from family and friends and might therefore manage without the use of a DHA-MD.

Stigmatization and shame related to the psychological condition are other important factors influencing GPs’ decision to prescribe DHA-MD. The majority of GPs reported that they would prescribe DHA-MD to patients who struggle with stigmatization and shame, which aligns with findings from an international systematic review [53] and a qualitative study from Austria [54] on perceived barriers and facilitators of mental health service utilization, both of which identified shame and fear of stigmatization as major patient-related barriers to seeking treatment. A possible explanation could be that DHA-MD are available independent of time and place [55] providing a certain degree of anonymity when using it. Therefore, GPs may perceive them as a promising option for these patient groups, overcoming their perceived barriers for treatment.

The time- and location-independent usability of DHA-MD might also explain why GPs would particularly prescribe them to patients with limited geographical mobility. This is consistent with the survey by Dockweiler et al. [56], analyzing online interventions for depression from the perspective of medical practitioners and therapists, showing agreement regarding the use for patients living in rural areas.

#### Indication area, clinical status and therapy experience

Beyond these patient-related factors, GPs also take clinical aspects into account when deciding whether to prescribe DHA-MD, in particular the indication and disease severity. The interviews revealed that GPs would prescribe DHA-MD to patients with anxiety and panic disorders, depressive disorders, adjustment disorders, or insomnia. One possible explanation could be that available DHA-MD are grounded in the scientific principles of cognitive behavioral therapy [18, 57], whose effectiveness via online-formats has been well established across several indications, such as depression [58] and insomnia [59].

Our results show that GPs would primarily prescribe DHA-MD to patients with mild to moderate mental disorders, emphasizing the importance of a certain degree of clinical stability. The reason for this might be that GPs often feel insufficiently informed about the contents and functionalities of the apps [27, 42] and do not feel sufficiently involved in the treatment with the apps [23, 27], which may lead them to prescribe them predominantly to patients with less severe conditions. This might also explain the clear reluctance to prescribe DHA-MD in our study to patients with multiple mental health conditions, as GPs might perceive them as too severely burdened to use a DHA-MD. In this context, the distinction between guided and unguided DHA‑MD may be particularly relevant. DHA‑MD that include professional guidance, such as regular feedback or support by trained professionals (e.g. as implemented in apps like those provided by ‘HelloBetter’ [60]), can be beneficial across symptom severity levels and may therefore also be offered to patients with more severe symptoms, as guided formats have the potential to enhance adherence and treatment effectiveness [61]. However, limited awareness of the contents and functionalities of DHA-MD among GPs, along with limited time to familiarize themselves with these apps, may currently restrict a differentiated use of guided versus unguided DHA-MD in routine care. This highlights the need for concise, easily accessible information resources that summarize key contents, functionalities, and indications of available DHA‑MD, enabling GPs to make informed choices despite time constraints in routine practice.

#### Treatment priority and purpose

In our study, GPs reported that they would prescribe DHA-MD to support in-person care or to bridge waiting times for psychotherapy, which is in line with the study by Weitzel et al. [27]. They examined the current use of e-mental health by healthcare professionals in Germany, indicating that GPs surveyed would predominantly prescribe e-mental health interventions, including DHA, primarily to bridge waiting periods and as supplementary self-help for patients. This was strengthened by the qualitative study of Sarradon-Eck et al. [26], which analyzed GPs’ attitudes towards prescribing mHealth apps in France, stating that GPs view these apps as a complementary extension of routine care - particularly useful for promoting self-monitoring, enhancing patient education and improving treatment adherence. Heterogeneity was expressed in our study regarding the use of DHA-MD as a follow-up treatment. In the cross-sectional survey by Weitzel et al. [56], none of the GPs supported the use of e-mental health interventions as aftercare. Our interviews could provide a possible explanation for this finding, particularly in the context of DHA, as GPs emphasized that patients who are already familiar with basic therapeutic strategies taught in psychotherapy might gain only limited additional benefits, given that DHA-MD are often based on similar scientific principles. In contrast, first-time users were perceived to benefit substantially more.

There was a clear reluctance among participating GPs to use DHA-MD as a primary treatment method, which is consistent with a survey conducted by a German health insurance company on motives and circumstances for prescribing DHA [62].

### Strengths and limitations

Our study offers valuable insights that contribute to a comprehensive understanding of the use and benefits of DHA-MD for patients with mental disorders in primary care from the perspective of GPs. By employing a mixed-methods approach, we were able to combine quantitative data, which highlight broader trends, with qualitative data that provide deeper insights into individual GP perspectives. This methodological approach ensures that our findings are relevant both for clinical practice and for other healthcare stakeholders (e.g., DHA manufacturers or BfArM). Our anonymous online survey may be biased by self-selection, potentially attracting participants with prior interest or strong opinions about DHA-MD, which could lead to an overestimation of both positive and negative attitudes or engagement with these digital interventions. The qualitative interviews predominantly involved already interested GPs. However, the mixed-methods design helped to address these limitations. The integration of quantitative and qualitative data allowed for mutual validation and supplementation, reducing method-specific biases and enhancing the robustness and practical relevance of the overall findings. With regard to generalizability, it is important to acknowledge that our findings are embedded within the specific context of DHA in Germany. The German DHA framework provides a distinct legal foundation for the prescription and reimbursement of such applications. Nonetheless, the results may also hold relevance for other countries that have recently implemented comparable initiatives, including France with the transitional coverage for digital health applications (PECAN) [16], Belgium with the mHealth validation pyramid [14], and the United Kingdom with the National Health Service Apps Library [15]. However, differences between national healthcare systems and reimbursement structures may constrain the direct transferability of our findings. Consequently, further international research is warranted to explore whether the patterns identified in our study can be replicated in other healthcare contexts.

## Conclusion

In conclusion, our study provides initial insights into GPs’ assessments of which patients they deem suitable for DHA-MD and the treatment contexts in which they would prescribe them in primary care. In making these decisions, they consider each patient’s individual skills, clinical condition, motivation, and likelihood of engaging with the app. As reflected in the high approval rates in our study, especially younger patients, patients with a high level of motivation and digital affinity, with limited mobility, as well as with primarily mild to moderate mental disorders are considered suitable for the use of a DHA-MD. Moreover, GPs primarily prescribe DHA-MD as a temporary solution to bridge waiting times for psychotherapy appointments or as a supplementary therapy option, rather than as standalone intervention. At the same time, this pre‑selection by GPs may carry the risk that certain patient groups are systematically excluded from using a DHA‑MD. To mitigate this risk, shared decision‑making in GP consultations may help assess whether individual patients would benefit from a DHA‑MD, taking into account patients’ capabilities, such as digital literacy. Beyond this, the provision of structured support (e.g. through a specially trained medical assistant embedded in the GP practice) and efforts to strengthen digital health literacy at the population level may further reduce digital inequalities and promote more equitable access to DHA‑MDs.

## Supplementary Information

Below is the link to the electronic supplementary material.

Supplementary Material 1

Supplementary Material 2

Supplementary Material 3

## Data Availability

The datasets generated during the current study are available from the corresponding author on reasonable request.

## Abbreviations

BfArM: German: Bundesinstitut für Arzneimittel- und Medizinprodukte
DHA: Digital Health Application
DHA-MD: Digital Health Applications for Mental Disorders
EU: European Union
GP: General Practitioner
GRAMMS: Good Reporting of a Mixed Methods Study
MDR: Medical Device Regulation
mHealth: Mobile Health Applications
