# Supplementary material for: Exploring general practitioners’ perspectives on the use and benefits of digital health applications for mental disorders in primary care: a mixed-methods study
Source: BMC Health Serv Res. 2026 Feb 28;26:379. doi: 10.1186/s12913-026-14256-0 (PMC13011575; doi:10.1186/s12913-026-14256-0)
Supplement: Supplementary file 1 — Supplementary Material 1 [file 12913_2026_14256_MOESM1_ESM.docx]

### Additional file 1: Results of interviews with General Practitioners

| **Main category** | **Subcategory** | **Example quote** |
| --- | --- | --- |
| **Suitable patient groups** | - **Indication area** - **Disease severity** - **Healthcare needs** - **Personal characteristics** | *“So, patients with an adjustment disorder or a mild depressive episode. They handle it well.” (GP_ DD06*)*  *“So, patients with mild depressive disorders or moderate depression might cope better than those with severe depression because their drive is often so limited that they may not have the motivation to engage with it on their own. Or to use this tool appropriately.” (GP_DD05)*  *“[…] So, if someone says, 'No, I have a good relationship with the psychotherapist, I've been to him before, I'll be fine there,' then I wouldn't want to persuade them to get a DHA. That's clear.” (GP_FFM01*)*  *“Mhm. Yes, I think that's probably the keyword 'that they could use it'. Of course, for some people it just somehow fails because of their affinity, so to speak, for the device, which is connected to an app like this.” (GP_FFM04)*  *“So yes, digital literacy and motivation. […] And openness to new forms of treatment in any case. And of course, the desire to actively work on their health themselves.” (GP_ DD05)* |
| **Unsuitable patient groups** | - **Indication area** - **Patients who already had General practitioner/ psychotherapist treatment** - **Disease severity** - **Personal characteristics** - **Social circumstances** | *“And all these things that are unstable, meaning everything associated with psychoses—so, anything related to psychotic disorders or substance use disorders—except for smoking and mild or risky alcohol consumption. But everything involving psychotropic substances is probably rather difficult. So, when it goes in the direction of drug use. Psychotic symptoms. Suicidality. That’s basically all ruled out, I think.” (GP_FFM04)*  *“And then there’s always the question of what exactly the patient has and to what extent. In my experience, if patients have already undergone psychotherapy, especially cognitive behavioral therapy, and they are somewhat familiar with it, then the content in the app is usually not as beneficial because they already have some basic knowledge—like, "What can I do in general?" They have typically learned that in their previous therapy. So, they don’t gain as much from it. Whereas if someone is dealing with a psychological issue for the first time and is still quite new to it, they might benefit from it significantly more.” (GP_FFM03)*  *“Yes, exactly. A severe depression, of course, should rather be treated in a hospital than with a DHA. Especially when motivation is reduced, I think it’s important to carefully consider what is the one task the patient can still manage—and what is the most important one. Is it more important for them to download the DHA, or is it more crucial that they secure a spot in psychotherapy? You really have to prioritize and assess what the patient is still capable of. Yeah. But exactly, I would be cautious in that regard.” (GP_FFM02)*  *“No. If someone is insecure or has a highly anxious personality, they won’t do it either because they’re too uncertain—like, "What happens to my data? Can I even do this?" and so on. Things like that. That just can’t be the case.“ (GP_ DD06)*  *“And patients who work a lot on the computer during the day and are actually good at using such technical devices have also given feedback, saying, "I'm already on the computer all day, and then I spend even more time on my phone or tablet because I have to enter my data there." (GP_DD02)*  *“So, I definitely wouldn’t prescribe it to people who can no longer manage it independently. That means everyone in a nursing home—or, well, not necessarily all nursing home residents, but if someone needs a caregiver to participate in something like this, that would be quite a significant hurdle for me. Not an absolute one, but a relatively high one, because they would always need someone else just to set it up and manage it. So, people need to be able to operate their smartphone independently.” (GP_FFM02)* |
| **Advantages for patients** | - **Bridging waiting times for psychotherapy** - **DHA-MD as initiation and support of in-person care** | *“[…]. Above all, the DHA-MD also fill a gap, because it often takes a very long time here to get an appointment with a psychologist or a psychiatrist. And the patients can then use the time when they are left hanging a bit to bridge it with this. And they have the feeling that they can already do something themselves for their recovery or to feel better. And it is also something for me that I can give them along the way, which also gives me the opportunity to do something for the patients." (GP_DD05)*  *“So, one advantage is, as already mentioned, bridging the long waiting time until psychotherapy can begin, which can often take several months. I can support the medication treatment. And also, the psychiatric treatment.” (GP_FFM03)*  *“Then the entry into psychotherapy in general, which I mentioned earlier. So I think it can make that easier.“ (GP_DD08)* |

*Pseudonymization codes
