# Supplementary material for: Exploring general practitioners’ perspectives on the use and benefits of digital health applications for mental disorders in primary care: a mixed-methods study
Source: BMC Health Serv Res. 2026 Feb 28;26:379. doi: 10.1186/s12913-026-14256-0 (PMC13011575; doi:10.1186/s12913-026-14256-0)
Supplement: Supplementary file 2 — Supplementary Material 2 [file 12913_2026_14256_MOESM2_ESM.docx]

### Additional file 2: Integration of quantitative and qualitative findings

| **Main category** | **Subcategory** | **QUAN (Online Survey)** | **QUAL (Telephone Interviews)** | **Integration** |
| --- | --- | --- | --- | --- |
| Age Group | Patients < 18 years | Most GPs (87.0 %) would not prescribe DHA-MD. | GP expressed disappointment about the regulatory age restriction; especially younger patients with mental health issues could benefit from such tools. | *Divergence/Complementarity:* Most GPs refuse to prescribe DHA-MD due to regulations; qualitative data suggest openness if regulations allowed it. |
|  | Younger or middle-aged patients  (18-65 years) | Most GPs would prescribe DHA-MD to younger (18-35 years; 79.6 %) or middle-aged patients (36-65 years; 76.2 %). | Younger to middle-aged patients (up to < 60 years) are considered suitable, as this age group tends to have the necessary digital affinity, openness, and motivation to engage with the contents of the DHA-MD. | *Convergence:*  Both strands emphasize younger/middle-aged patients as most suitable for DHA-MD use. |
|  | Seniors or very elderly (> 66 years) | GPs would not prescribe DHA-MD to seniors (66-80 years; 76.0 %) or very elderly (> 80 years; 95.2 %). | Older adults (> 75 years) often face usability challenges with digital technologies, and may lack the necessary familiarity and confidence in their use. | *Convergence:*  Both strands identify older adults as unsuitable for DHA-MD use. |
| Psychosocial Burden and Social Resources | Patients with problems in interpersonal relationships | Heterogeneous responses: 53.4 % would prescribe, 46.6 % would not prescribe DHA-MD. | Not addressed. | *Unique quantitative finding.* |
|  | Patients with a stable social network | Heterogeneous responses: 57.8 % would prescribe, 42.2 % would not prescribe DHA-MD. | Not addressed. | *Unique quantitative finding*. |
|  | Patients with psychological problems they feel ashamed of | Most GPs (74.8 %) would prescribe DHA-MD. | Not addressed. | *Unique quantitative finding*. |
|  | Patients with pronounced fear of stigmatization | Most GPs (62.1 %) would prescribe DHA-MD. | Not addressed. | *Unique quantitative finding.* |
|  | Patients with lack of daily structure | Heterogenous responses: 53.8 % of GPs would prescribe, while 46.2 % would not prescribe DHA-MD. | GPs noted that a basic daily structure is needed for successful DHA-MD use; patients with a lack of daily structure are unlikely to engage with the DHA-MD. | *Complementarity:*  This heterogeneity is complemented by qualitative findings indicating that the lack of a daily structure can hinder effective use, which could explain the reluctance of some GPs. |
|  | Patients who struggle to access psychotherapy | Heterogenous responses: 59.9% would prescribe, 40.1% would not prescribe DHA-MD. | Not addressed. | *Unique quantitative finding.* |
| Indication area | - | Not addressed. | Suitable: Anxiety/panic disorders (easier access, reinforcement of relaxation techniques), depression (controversially discussed: easier access vs. risk of reduced adherence/social withdrawal), adjustment disorders, stress-related complaints, burnout, psychosomatic conditions, insomnia, certain substance use disorders (e.g., smoking, risky alcohol consumption). Unsuitable: psychotic disorders with suicidality, PTSD, drug addiction. | *Unique qualitative finding.* |
| Clinical status and therapy experience | Patients who have already undergone psychotherapy | Heterogeneous responses:  54.2% would prescribe, while 45.8% would not prescribe DHA-MD. | GPs emphasized that patients who are already familiar with basic therapeutic strategies can only derive limited additional benefit. In contrast, first-time users can benefit significantly more. | *Complementarity:*  The heterogeneity can be explained by the perceived lower benefit for patients who have undergone therapy before and the higher benefit for first-time users. |
|  | Patients who have already positive experiences with DHA-MD | Most GPs (84.2 %) would prescribe DHA-MD. | Not addressed. | *Unique quantitative finding.* |
|  | Patients with multiple mental disorders | Most GPs would not (80.7 %) prescribe DHA-MD. | Not addressed. | *Unique quantitative finding.* |
|  | Patients with mild to moderate mental disorders | Most GPs (71.9 %) would prescribe DHA-MD. | One group of GPs considered DHA-MD suitable for patients with mild to moderate mental disorders, emphasizing the importance of clinical stability and the likelihood of sufficient motivation to engage with the content. | *Convergence:*  Both strands support the suitability of DHA-MD for patients with mild to moderate symptoms. |
|  | Patients with severe mental disorders | Not addressed. | Heterogenous views: Some GPs saw patients with severe mental disorders as unsuitable for DHA-MD use due to low drive and poor daily structure. Others considered DHA-MD suitable in stable phases, especially when in-person therapy was unlikely. | *Unique qualitative finding*. |
|  | Patients with an acute need for treatment | Heterogeneous responses:  45.1 % would not prescribe DHA-MD, while 54.9 % would prescribe. | Not addressed. | *Unique quantitative finding.* |
|  | Patients with a lack of motivation for outpatient psychotherapy | Heterogeneous responses: 41.1 % would prescribe DHA-MD, while 58.6 % would not prescribe. | Not addressed. | *Unique quantitative finding.* |
| Language skills | Patients with good knowledge of German  Patients with limited knowledge of German. | Most GPs (73.1 %) would prescribe DHA-MD.  Most GPs (71.7 %) would not prescribe DHA-MD. | GPs noted that language barriers can hinder the prescription of DHA-MD, especially when patients do not understand the purpose or contents. They emphasized that a lack of multilingual options for some apps limits their usability. | *Convergence:*  Both strands show that limited language skills reduce the likelihood of prescribing, with qualitative data underscoring the role of comprehension and availability of multilingual app versions. |
| Mobility | Patients with limited geographical mobility | Most GPs (78.6 %) would prescribe DHA-MD. | Not addressed. | *Unique quantitative finding.* |
|  | Patients with limited physical mobility | Most GPs (76.9 %) would prescribe DHA-MD. | Not addressed. | *Unique quantitative finding.* |
| Treatment priority and purpose | Primary treatment method | Most GPs (81.1 %) would not prescribe DHA-MD. | GPs considered DHA-MD suitable only as an accompanying treatment method, not as a primary treatment method. | *Convergence:*  Both strands indicate low willingness to prescribe DHA-MD as a primary treatment method. |
|  | Bridging waiting times for psychotherapy | Most GPs (83.8 %) would prescribe DHA-MD. | GPs reported that DHA-MD can fill the gap while patients wait for psychotherapy appointments and enable them to offer immediate support. | *Convergence:*  Both strands emphasize the role of DHA-MD in bridging therapy waiting times, highlighting benefits for continuous support. |
|  | As an accompanying treatment to psychotherapy | Most GPs (65.8 %) would prescribe DHA-MD. | GPs found that DHA-MD can support pharmacological or psychiatric treatments, especially during long waiting periods before the start of psychotherapy. | *Complementarity:*  Quantitative data reveal heterogeneity, while qualitative findings highlight the role of DHA-MD as supportive care alongside other treatments and during waiting times. |
|  | As a follow-up treatment to psychotherapy | Heterogeneous responses: 58.8 % would prescribe DHA-MD, while 41.2 % would not prescribe. | GPs emphasized that patients who are already familiar with basic therapeutic strategies can only derive limited additional benefit. In contrast, first-time users can benefit significantly more. | *Complementarity*:  The heterogeneity can be explained by the perceived lower benefit for patients who have undergone therapy before and the higher benefit for first-time users. |
|  | As an attempt to encourage initiation of psychotherapy | Most of GPs (63.9 %) would not prescribe DHA-MD. | Some GPs saw DHA-MD as a potential first step to lower barriers - such as shame - towards starting psychotherapy. | *Divergence:*  While quantitative data show limited support, qualitative findings reveal that some GPs view DHA-MD as a tool to reduce psychological barriers and motivate initiation of psychotherapy. |
| Digital literacy and usage habits | Digital affinity | Not addressed. | GPs stressed that digital affinity is essential for DHA-MD use. Patients need interest in digital technology, basic technical skills, device access, and the ability to download and activate apps independently. Positive prior app experiences may support usage. | *Unique qualitative finding.* |
|  | Problematic/ increased smartphone use | Not addressed. | GPs have expressed concerns about prescribing DHA-MD to patients with problematic smartphone use and consider this a relative contraindication. The decision to prescribe DHA-MD should be made on an individual basis and carefully considered in each case. | *Unique qualitative finding.* |
| Personality traits | Anxious personality | Not addressed. | GPs reported that patients with a generally anxious personality may be reluctant to use DHA-MD due to concerns about data security. | *Unique qualitative finding.* |
|  | Motivation for behavior change | Not addressed. | GPs highlighted that patients should be willing to actively work on their health, should have a high level of compliance, and feel sufficiently distressed to seriously strive for a change in behavior. They reported that patients should be open-minded to this innovative new form of treatment. | *Unique qualitative finding.* |
