# Supplementary material for: Exploring general practitioners’ perspectives on the use and benefits of digital health applications for mental disorders in primary care: a mixed-methods study
Source: BMC Health Serv Res. 2026 Feb 28;26:379. doi: 10.1186/s12913-026-14256-0 (PMC13011575; doi:10.1186/s12913-026-14256-0)
Supplement: Supplementary file 3 — Supplementary Material 3 [file 12913_2026_14256_MOESM3_ESM.docx]

### Additional file 3: Interview guide for General Practitioners

| **Introduction** | **Topic/Aim** |
| --- | --- |
| Good afternoon, Ms./Mr. ... I am very pleased that we were able to arrange this interview with you today.  Let me briefly introduce myself… | Welcoming the participants, thanking them for their willingness to participate, introducing yourself |
| In this interview, I would like to give you the opportunity to talk about your previous experiences with “apps of prescription.” We are also interested in your assessment of Digital Health Applications (DHA) for the treatment of mental health conditions (DHA-MD). | Aim/introduce the topic of the interview |
| Please feel free to speak openly. There are no “right” or “wrong” answers. What matters are your personal experiences and opinions. We would like to set aside approximately 30 minutes for this interview. With your consent, we would record the interview. This will allow me to concentrate better on our conversation and will make it easier for us to evaluate the results. | Procedure/there is no right or wrong, your opinions and experiences are important, duration of the interview approx. 30 min., recording |
| Clarification of questions from participants. | Questions |
| >> Turning on the recording device << | |
| **Question** | **Topic/Aim** |
| **1. Open introduction** | |
| - **What** is the first thing that comes to your mind when you think of Digital Health Applications (DHA), also known as “apps on prescription”? | Open introduction |
| **2. Experiences with Digital Health Applications (DHA)** |  |
| - **Did you already prescribe DHA in your practice?**   *If DHA have already been prescribed:*   - - For which indication areas have you prescribed DHA?   - Which specific DHA were they? Can you tell me the name?   - What proportion of these were DHA for mental health conditions (DHA-MD)?   *(If needed, explain which indications comprise DHA-MD)*   - - Which DHA-MD have you prescribed?   - Why did you choose to prescribe particularly these DHA-MD?   *If DHA have not yet been prescribed:*   - - Which DHA are you familiar with?   - Why have you not prescribed any DHA so far?   🡪 *Then continue with section* ***3. Attitudes with DHA-MD*** | Prescription frequency  (Dis)advantages of specific DHA-MD  Different care models (e.g., self-management; involvement of GP (e.g., feedback, monitoring)) |
| - **What experiences** have you generally had with prescribing DHA-MD in primary care? | Notes on general attitudes towards/experiences with DHA-MD |
| **3. Attitudes towards DHA-MD** |  |
| After discussing your personal experiences with DHA-MD, I would now like to talk with you about DHA-MD in primary care more generally.   - **What significance** do DHA-MD have in primary care for patients with mental disorders? - What **advantages** could they offer patients? - What **disadvantages** could they offer patients? - Which **groups of patients manage well** with DHA-MD, and why? - Which **groups of patients do not manage well** with DHA-MD, and why? | (Dis)advantages for patients |
| - **How** do you assess **the need** for DHA-MD in primary care for patients with mental disorders? | DHA-MD as a treatment option necessary/not necessary, sufficient apps available, expansion of care structures needed, etc. |
| - In your opinion, what **characteristics** should **patients** have in order to be able to use a DHA-MD? - Indication area and disease severity - Personality traits - Social conditions/circumstances - (Level of) care need | (Un)suitable patient groups (with regards to indication area), personal factors, social factors |
| - **How** do you assess the **effectiveness** of DHA-MD for patients?   - How do you assess the effect on psychological symptoms?   - How do you assess the benefit of bridging the waiting period for psychotherapeutic treatment?   - How do you assess its usefulness in terms of managing patients own mental disorder?   - To what extent does the effectiveness of DHA-MD differ for the same indication area?   - To what extent does the effectiveness of DHA-MD differ across different indication areas? | (Dis)advantages compared to traditional psychotherapy, opportunities/potentials of DHA-MD, risks for patients |
| **4. Implementation factors and framework conditions for DHA-MD** |  |
| - **What opportunities** do you see for yourself as a general practitioner in relation to the use of DHA-MD in patient care?   - What opportunities do you see for healthcare in your region? - **What opportunities** do you see for the **cooperation** **between general practitioners and psychotherapists**? | Promoting factors (prescribers' perspective), care structures, cooperation between general practitioners and psychotherapists |
| - **What risks** do you see for yourself as a general practitioner in relation to the use of DHA-MD in patient care? | Unresolved liability issues, importance of personal care |
| - What **challenges** do you see in relation to the use of DHA-MD in **patient care**? - Do you have any **ideas for improvements**? What would be needed to achieve this? (Perhaps also on the part of legislators, e.g., more education and information about DHA) | Lack of information, unclear remuneration structure  In some cases, no remuneration for consultation |
| - So that you can trust DHA-MD: **Which criteria** do you consider essential/important for **approval**? - **What conditions** must be met for you to prescribe DHA-MD? | Data protection, proof of effectiveness, ease of use, clarification of cost coverage, clarification of liability |
| - What are **your hopes for the future development** of DHA for people with mental health conditions? | Future/outlook |
| **5. Conclusion** |  |
| - In your opinion, have I forgotten anything else on this topic? - Is there anything else that is on your mind/that you would like to share with us? |  |
